# The association between treated psychiatric and neurodevelopmental disorders and out-of-home care among Finnish children born in 1997

**DOI:** 10.1007/s00787-021-01819-1

**Published:** 2021-06-08

**Authors:** Antti Kääriälä, David Gyllenberg, Reijo Sund, Elina Pekkarinen, Markus Keski-Säntti, Tiina Ristikari, Tarja Heino, Andre Sourander

**Affiliations:** 1 Finnish Institute for Health and Welfare (THL), Helsinki, Finland; 2 Faculty of Social Sciences, University of Helsinki, Helsinki, Finland; 3 Research Centre for Child Psychiatry and INVEST Research Flagship, University of Turku, Turku, Finland; 4 Department of Adolescent Psychiatry, Helsinki University Hospital, Helsinki, Finland; 5 Institute of Clinical Medicine, University of Eastern Finland, Kuopio, Finland; 6 Office of the Ombudsman for Children in Finland, Jyväskylä, Finland; 7 Finnish Institute for Health and Welfare (THL), Oulu, Finland; 8 Itla Children’s Foundation, Helsinki, Finland; 9 Department of Child Psychiatry, Turku University Hospital, Turku, Finland

**Keywords:** Psychiatric disorders, Neurodevelopmental disorders, Self-harm, Child welfare, Out-of-home care

## Abstract

Comprehensive overviews of the use of psychiatric services among children and adolescents placed in out-of-home care (OHC) by child welfare authorities are scarce. We examine specialized service use for psychiatric and neurodevelopmental disorders among children and adolescents in a total population involving children in OHC. We used the longitudinal administrative data of a complete Finnish birth cohort 1997 (*N* = 57,174). We estimated risk ratios (*RR*s) for a range of diagnosed psychiatric and neurodevelopmental disorders among children in OHC. We also estimated RRs for OHC among those with diagnosed disorders. We used descriptive methods to explore the timing of first entry into OHC relative to the first diagnosis. Among children in OHC, 61.9% were diagnosed with any psychiatric or neurodevelopmental disorder, compared with 18.0% among those never in OHC (*RR*: 3.7; 95% CI 3.6–3.8). The most common diagnosed disorders among children in OHC were depression and anxiety disorders, neurodevelopmental disorders, and oppositional defiant disorder/conduct disorder (ODD/CD). Among all children with any diagnosis, 18.1% experienced OHC, compared with 2.5% among those without a diagnosis (*RR*: 7.4; 95% CI 6.9–7.9). Of those diagnosed with self-harm and suicidality, ODD/CD, substance-related disorders, and psychotic and bipolar disorders, 43.5–61.2% experienced OHC. Of the children in OHC receiving psychiatric services, half were diagnosed before first placement in OHC. The majority of children with experience in OHC were diagnosed with psychiatric or neurodevelopmental disorders. They comprised a significant proportion of individuals treated for severe and complex psychiatric disorders and self-harm.

**Supplementary Information:**

The online version contains supplementary material available at 10.1007/s00787-021-01819-1.

## Introduction

According to a global estimate, approximately 2.7 million children live in out-of-home care (OHC) due to their need for child welfare [1], which aims at securing children’s health and development in the presence of adverse circumstances. Children placed in OHC are known to have high need and expenditure for, and high use of, mental health services [2–16], with several studies highlighting the importance of timely provision of evidence-based interventions [10, 17, 18]. To facilitate the service planning of psychiatric services for children in OHC and child welfare services for children in psychiatric care, it is essential to have overarching knowledge of the concomitant use of these two services, as well as of pathways from one service to another. This knowledge is instrumental for assessing the appropriate type, quantity, and timing of the psychiatric interventions required in child and family health and social services.

In a recent meta-analysis, the prevalence of current psychiatric disorder was 49% among children in OHC [2]. According to our literature review (see Supplement text and Supplement table 1), placement in OHC has been associated with several types of childhood psychiatric disorders, including depression and anxiety [2, 4, 5, 7, 9, 13–16], oppositional defiant disorder/conduct disorder (ODD/CD) [2, 2, 4, 4, 5, 14–16, 9], post-traumatic stress disorder [2, 10], substance use disorders [4, 10, 13, 14, 20, 21], eating disorders [13, 14], and suicidal behavior [14, 22]. Placement in OHC has also been associated with neurodevelopmental disorders, such as attention-deficit/hyperactivity disorder (ADHD) [2, 4, 5, 7, 9, 14–16, 19] and learning disorders [4, 7, 16]. One Danish study has compared specialized psychiatric service use longitudinally, up to age 11, between children in OHC and children never in OHC. Of the children in OHC, 20% had a psychiatric diagnosis compared with 3% among children never placed in OHC [5]. Existing evidence on service use due to psychiatric disorders is, however, mostly based on cross-sectional studies, small samples, or local service programmes, often without a comparison group. In addition, while the abovementioned studies have focused on psychiatric outcomes among children in OHC, reports on placements into OHC among children treated for psychiatric disorders are rare [8, 23]; such information is instrumental for assessing the need for social work within psychiatric clinics and vice versa. We are also not aware of any studies that investigate the temporal relation between entries into OHC and psychiatric services, which is important for understanding what kind of expertise is needed in both social and health services, as well as for identifying service pathways and the timing of the service use. In summary, evidence concerning the association between the need for psychiatric care and support by child welfare is insufficient.

To address these gaps in the evidence, we provide a comprehensive overview of psychiatric health service use and child welfare’s OHC placements up to the age of 18 among a complete Finnish national birth cohort born in 1997. To examine the use of these two services bi-directionally, we first assessed the likelihood of specialized health service use among children placed in OHC in comparison with those never placed in OHC. In doing so, we investigated a range of diagnosed psychiatric and neurodevelopmental disorders and their comorbidity. Second, we assessed the likelihood of individuals with these diagnoses being placed in OHC. Based on previous findings, we expected that a range of diagnoses would be associated with OHC. Additionally, we examined the timing of first placement in OHC in relation to the first diagnosis of the investigated psychiatric and neurodevelopmental disorders.

## Methods

### The study design and study population

We used data from the 1997 Finnish Birth Cohort study. Managed by the Finnish Institute for Health and Welfare (THL), the birth cohort contains data from several nationwide registers for all children born in Finland during that year. We excluded individuals who emigrated or died before turning 18. We followed all the included cohort members through registers from birth to their 18th birthday (i.e. 1997–2015) for entries into OHC and investigated psychiatric and neurodevelopmental disorder diagnoses up to that date. We followed the reporting recommendations of the Strengthening the Reporting of Observational Studies in Epidemiology (STROBE) guidelines for cohort studies (Supplement table 2).

### Data sources

The personal identity codes assigned to all Finnish residents enable the accurate linkage of data from different registers into a single dataset. We used data provided by the Medical Birth Register [24] for the identification of the children born in Finland in 1997, their date of birth, and sex. In addition, we gathered data from the Child Welfare Register for placements in OHC and from the Care Register for Health Care [25] for the dates of inpatient and outpatient visits to public specialized hospital clinics and their accompanying diagnoses. Statistics Finland [24] provided data for dates and causes of death [26] and the Digital and Population Data Services Agency [24] provided data on emigration and residential location.

Maintained by the THL, the Child Welfare Register involves records of all children placed in OHC in Finland. The records include information on the start and end dates of placements, placement settings (e.g. foster care and residential care), and the legal grounds of the placement decision. The register is gathered by mandated biannual reporting from the municipal child welfare authorities from 1991 onwards, thus covering our follow-up from 1997 to 2015. In the Finnish child welfare system, OHC is considered a last-resort measure, which is taken after in-home services have proven inappropriate, insufficient, or impossible. Municipal child welfare authorities make the decision to place a child in OHC if the parents and children agree with the measures and in emergency situations. Contested care orders are decided in an administrative court.

Data on visits to specialized health services are based on the Care Register for Health Care, maintained by the THL. The register includes records of the start and end dates of visits to any hospital inpatient unit or outpatient clinic in public hospitals, a mandatory primary diagnosis, and optional secondary diagnoses. Inpatient data are available from 1969 and outpatient data from 1998, meaning that the outpatient records are partly incomplete for the first year of the 1997 birth cohort. The register has been widely used in epidemiological research [25], including studies on the diagnostic validity of several disorders [27–29] and a study on the incidence of specialized psychiatric health care use in adolescents in the 1997 birth cohort [30].

### Measures

We defined experience of OHC as any placement in OHC before a person’s 18th birthday. This measure thus captures children in OHC for both the short and long term, including those who reunited with their families during the study period. We studied lifetime prevalence rates of psychiatric diagnoses, which we defined as an inpatient and outpatient specialized health service visit before the person’s 18^th^ birthday. For measurement, we investigated primary and secondary diagnoses according to the 10th revision of the International Classification of Diseases (ICD-10) for any psychiatric disorder (ICD-10 codes F10–F99) and separately according to the seven diagnostic classes specified in the Supplementary appendix (Supplement table 3); consequently, each person could be recorded under one or more of these specific diagnostic classes. In addition, to investigate the temporal relation between first placement and first diagnosis, we used age at first placement and age at first diagnosis as continuous variables.

### Statistical analysis

We examined cumulative probabilities (i.e. from birth to the age of 18) for visits to psychiatric outpatient clinics and inpatient care among children with experience of OHC, as well as cumulative probabilities for placement in OHC among children diagnosed with specified psychiatric and neurodevelopmental disorders by the age of 18. To quantify the association between placement in OHC and psychiatric service use, and vice versa, we computed crude risk ratios (*RR*s) with 95% confidence intervals (CIs). The RR was computed by dividing the cumulative probability in the exposed group by the cumulative probability in the unexposed group. We investigated the timing between the first placement and the first diagnosis using descriptive methods. We also completed several additional and sensitivity analyses (see Supplementary appendix). We conducted the analyses with R for Windows (version 3.6) [31].

## Results

There were 58,802 individuals in the Finnish Birth Cohort 1997 study who survived the perinatal period. After excluding those who had any history of emigration (*n* = 1370) or died (*n* = 258) before the end of the follow-up at the age of 18, the population comprised 57,174 individuals.

Of the study population, 3254 (5.7%) individuals experienced placement in OHC before the age of 18. The median (interquartile range) age at first placement was 12.7 years (5.58–15.1 years) (Table 1), with first placements peaking at infancy (age 0) and in adolescence (ages 14–15). The most typical placement settings were residential care (61.9% of those placed in OHC) and a foster family (19.5%) (Table 1), with foster families dominating among those placed in care for the long term at younger ages and residential care dominating among those placed for the short term and during adolescence (not shown in the table).

**Table 1:** Children in out-of-home care in the 1997 birth cohort by placement characteristics

	All children in out-of-home care
Sex, no. (%)	
Boys	1608 (49.4)
Girls	1646 (50.6)
Age at first placement, median (IQR)	12.7 (5.6–15.1)
Total length of time in care, days, median (IQR)	396 (62–1417)
Number of placements, median (IQR)	3 (1–5)
Most typical placement setting, no. (%)	
Foster family	634 (19.5)
Professional foster home	472 (14.5)
Residential care	2014 (61.9)
Other settings	134 (4.1)
Total, no.	3254

*IQR* inter quartile range

**Fig. 1:**
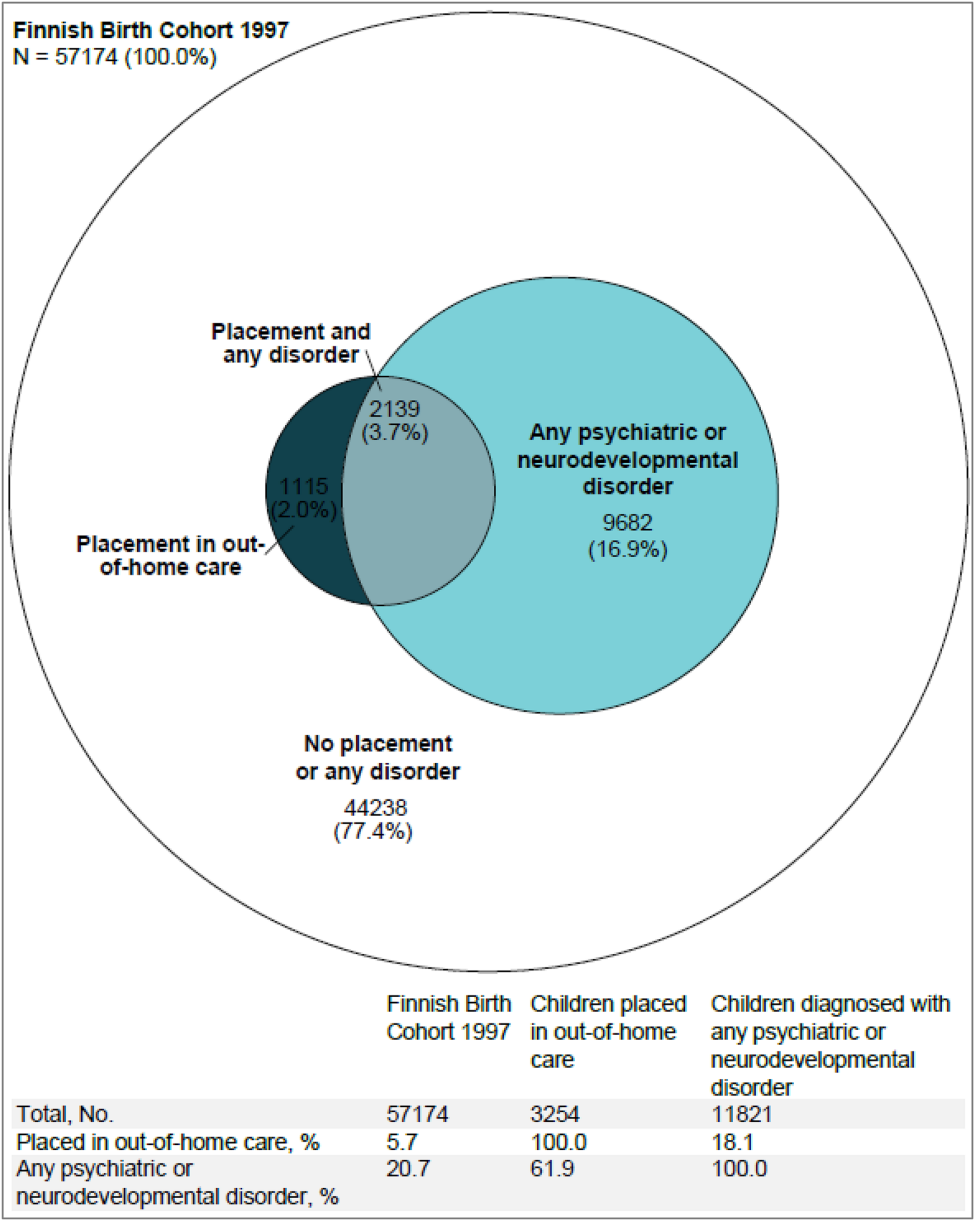
A Euler diagram of individuals in the Finnish Birth Cohort 1997 placed in out-of-home care and diagnosed with any psychiatric or neurodevelopmental disorder

We observed 11,821 (20.7% of the cohort) individuals who used outpatient or inpatient specialized services for any psychiatric or neurodevelopmental disorder before age 18 (Fig. 1). Of these children, 2139 (18.1%) had been placed in OHC, compared with 1115 (2.5%) of the 45 353 individuals with no diagnosis (*RR* of OHC: 7.4; 95% CI 6.9–7.9). Of those with experience of OHC, 61.9% had any diagnosis, compared with 18.0% among those never in OHC (*RR* of diagnosis: 3.7; 95% CI 3.6–3.8). Restricting the analysis to inpatient records alone suggests that children in OHC are even more disproportionally present in inpatient units than in outpatient clinics (Supplement text and Supplement Figs. 1 and 2). Sex-stratified analysis indicates that psychiatric service use due to any psychiatric or neurodevelopmental disorder concerns both sexes similarly (Supplement text and Supplement Figs. 3 and 4).

**Fig. 2:**
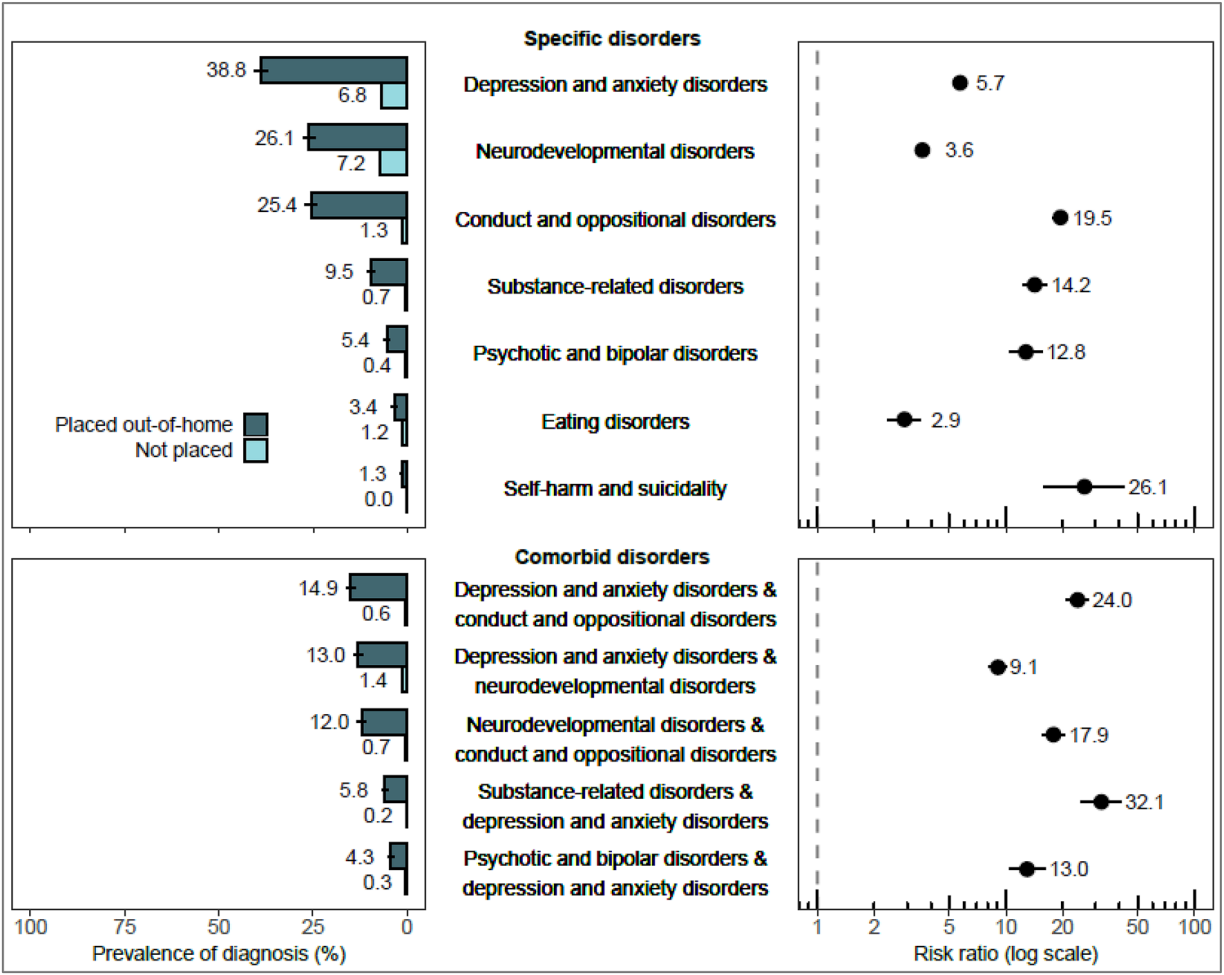
The risks of specific psychiatric or neurodevelopmental disorder diagnoses and the five most common comorbid diagnostic classes among children in out-of-home care compared with children never in out-of-home care

Children with experience of OHC were much more likely to use specialized services due to each of the specific disorders compared with those never placed in OHC (see Fig. 2). The most common diagnosed disorders among children in OHC were depression and anxiety disorders (38.8% vs. 6.8% among those never placed), neurodevelopmental disorders (26.1% vs. 7.2%), and ODD/CD (25.4% vs. 1.3%) (Fig. 2). Children with experience of OHC were also frequently diagnosed with comorbid combinations of these three most common diagnostic classes, with cumulative probabilities ranging from 12.0 to 14.9% (Fig. 2). Children in OHC had the highest RRs for self-harm and suicidality (*RR*: 26.1; 95% CI 16.0–42.7) and ODD/CD (*RR*: 19.5; 95% CI 17.7–21.4) (Fig. 2). Their RRs were also high or very high for most comorbid combinations.

**Fig. 3:**
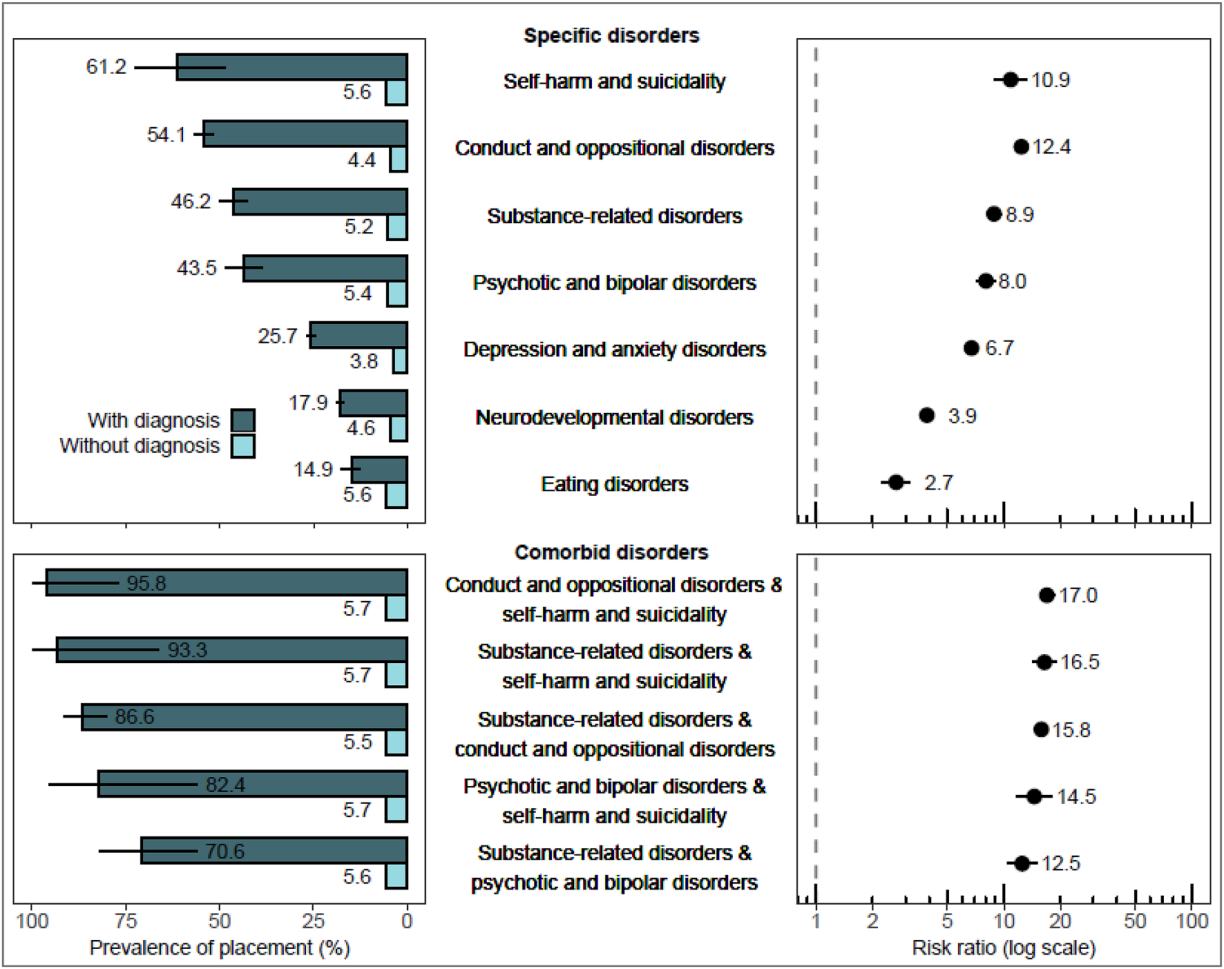
The risk of placement in out-of-home care among children with specific psychiatric or neurodevelopmental disorder diagnoses compared with children without such diagnoses, including five comorbid diagnostic classes with the highest proportion of placements

Those diagnosed with some of the specific disorders had high probabilities of having experience of OHC. More than half of the individuals diagnosed with self-harm and suicidality (61.2%) and ODD/CD (54.1%) experienced placement in OHC (Fig. 3). In addition, large percentages of children diagnosed with substance-related disorders (46.2%) and psychotic and bipolar disorders (43.5%) had experience of OHC. The likelihood of placement was further increased among those with comorbid diagnoses. Most notably, of those with ODD/CD and a comorbid self-harm and suicidality diagnosis, 95.8% had been in OHC.

**Fig. 4:**
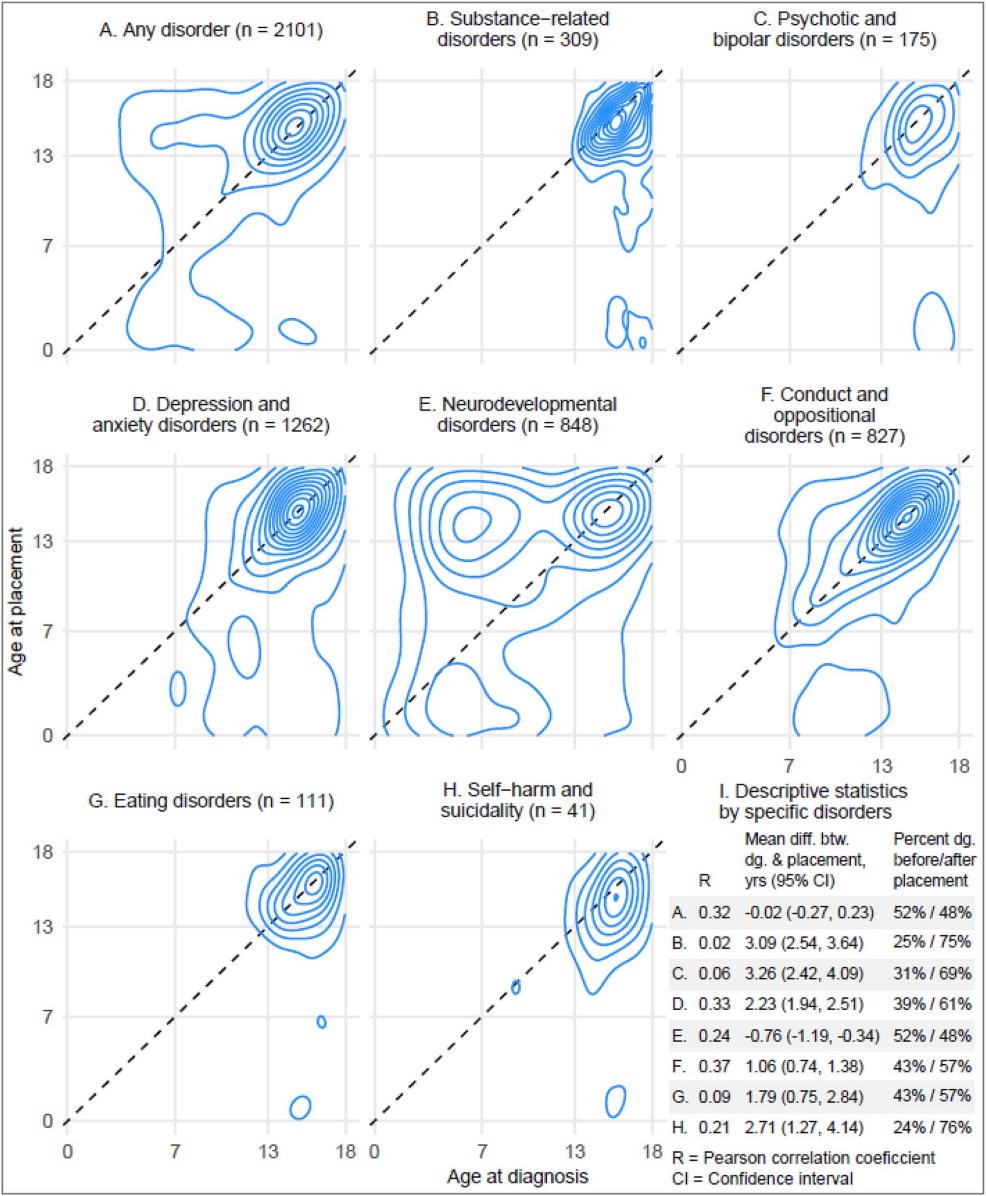
The timing of first placement into out-of-home care versus the timing of first diagnoses among those with experience of out-of-home care and diagnosed psychiatric or neurodevelopmental disorders

Age at first placement in OHC and age at first diagnosis of any psychiatric or neurodevelopmental disorder correlated moderately (*R* = 0.32) and occurred, on average, at the same time (mean time difference between first placement and first diagnosis: − 0.02 years; 95% CI − 0.27 to 0.23), with both events occurring in adolescence (Fig. 4). Approximately, half of the children who had both experience of OHC and a diagnosis of any disorder were diagnosed before the first placement. However, we observed differences in timing when examining specific disorders. ODD/CD diagnoses seemed to co-occur most closely with first placements in OHC (*R* = 0.37). In addition, substance-related disorders, and self-harm and suicidality were diagnosed after the first placement in three out of four cases.

Our results from the sensitivity analyses were similar to the main analysis (see Supplement text and Supplement Figs. 5–8).

## Discussion

The strengths of the present study are the total population coverage of children born in 1997 in Finland and the reliable nationwide register-based follow-up until age 18. The main results are, first, that the majority of children with experience in OHC were diagnosed with a psychiatric or neurodevelopmental disorder in specialized services during childhood. In addition, approximately one in five children with a diagnosed disorder experienced placement in OHC, compared with approximately 1 in 40 children without such a diagnosis. Most notably, the majority among those diagnosed with self-harm and ODD/CD experienced placement in OHC.

Our findings indicate that most children placed in OHC are treated for psychiatric or neurodevelopmental disorders, confirming previous evidence [2–16]. Accordingly, we noted significant differences in the likelihood of childhood and adolescence psychiatric service use between children who experienced placement in OHC and those who did not. Our findings are in line with previous research in showing that children with experience of OHC are frequently treated for depression and anxiety disorders, neurodevelopmental disorders, and ODD/CD [2, 4, 5, 7, 9, 10, 13–16, 19]. In addition, the prevalence of substance-related disorders in this study is approximately the same as that found by a review on the prevalence of lifetime alcohol and drug abuse among children in OHC [20]. The prevalence of self-harm and suicidality among children in OHC in this study was 1.3%, which is low compared with the prevalence of suicide attempts in a meta-analysis (3.6%) [22]. This difference may result from different ascertainment methods since the meta-analysis included self-reports while we used only inpatient and outpatient records.

This study add to the scarce literature on the experience of OHC among those receiving psychiatric services [8, 23]. Halfon et al. [8] used utilization data from California’s Medi-Cal programme from the 1980s, and Kiuru and Metteri [23] relied on data from one Finnish psychiatric hospital, whereas our study covers one full national birth cohort. We found that children in OHC comprised 18.1% of all children who received inpatient and outpatient services due to psychiatric and neurodevelopmental disorders, although they represented just 5.7% of the cohort. Halfon et al. [8] found that foster children were even more disproportionately present in psychiatric services, which could be explained by contextual differences between California and Finland. We noted that children with experience of OHC comprised very high proportions of those diagnosed with self-harm and suicidality, ODD/CD, substance-related disorders, psychotic and bipolar disorders, and various comorbid combinations of the investigated disorders.

There are several potential explanations for the over-representation of children with experience of OHC in psychiatric services. First, due to several reasons—such as a history of maltreatment, trauma, and genetic vulnerability—psychiatric disorders are more common among children in OHC than in the general population that is not in OHC, even before entering OHC [15]. Furthermore, a meta-analysis indicates that being in OHC has no effect on these children’s internalizing, externalizing, or total behavior problems [32], suggesting that elevated psychiatric needs among these children persist even while being placed in OHC. Second, evidence indicates that being in OHC facilitates access to psychiatric services [3, 6], which may result, for example, from the improved identification of needs or referrals by social services. Our findings also highlight the possibility that, vice versa, receiving psychiatric care may increase the likelihood of entering OHC. Third, OHC is targeted not only at maltreated and disadvantaged children, but also to children and young people whose behavior, such as criminality and substance use, puts them at risk. This is then directly related to some disorders, including ODD/CD and substance-related disorders, via diagnostic criteria if these individuals are referred to psychiatric services.

We also observed that age at first entry into OHC and age at first diagnosis of any psychiatric or neurodevelopmental disorder co-occurred to a moderate extent and occurred, on average, at the same age in adolescence. Increased psychiatric needs and service use during adolescence among children in OHC has also been noted by other studies [3, 7, 8, 15]. The accumulation of first diagnoses and placements in adolescence may result from earlier, possibly subthreshold or undiagnosed psychiatric problems as these strongly predict later adverse outcomes, including psychopathology and antisocial behaviors [33–35], which may instigate child’s placement into OHC. In addition, we noted some disorder-specific variance in the correlation between age at first diagnosis and age at first entry into OHC. Specifically, placement and diagnosis ages were most strongly correlated for ODD/CD, most likely indicating that placing a child in OHC is a typical response to severe behavioral disorders. However, the timing of service entries may inform us of service provision, even if the correlation between diagnosis and placement age is weak or non-existent. When we observed children who were both placed in OHC and treated in psychiatric services, we noted that approximately half of them were diagnosed before their first entry into OHC. On the one hand, this suggests that psychiatric and neurodevelopmental disorders are often already treated before entering OHC. For example, neurodevelopmental disorders are typically diagnosed before school age or at elementary school age, while a large proportion of placements in OHC among children with these types of disorders occur during adolescence. On the other hand, some disorders were mostly diagnosed after first entry into OHC. These included self-harm and suicidality, substance-related disorders, and psychotic and bipolar disorders, for which adolescence is the typical period of onset.

Our findings underscore, first, the necessity of mental health assessment among children who are referred to OHC. Specific attention should be given to self-harming behavior, and severe and complex behavioral and emotional disorders for those who enter OHC at school age and adolescence. In addition, to treat mental health among children who are placed in OHC, the OHC system requires adequate resources in terms of knowledge, skills, and capacity. This includes social workers and children’s carers who work with these children in foster families and institutional settings on a daily basis understanding mental and behavioral health. Children in OHC with specific disorders may also benefit from evidence-based interventions, including multidimensional treatment foster care targeted at children with behavioral disorders [36]. Given that half of children in OHC with diagnosed psychiatric and neurodevelopmental disorders in this study were diagnosed before entering OHC, preventive interventions form an important approach. Several elements of effective youth- and family-directed interventions have been identified [37], but the development of effective family support interventions for families at risk of a child’s OHC placement remains a challenge [38]. In Finland, there is promising evidence from a parent training intervention offered for parents of preschool children with disruptive behavioral problems [39]. However, the implementation of evidence-based interventions for young people is lacking in the country, particularly in primary care settings.

Strengths of our study include the large nationwide register data that enables us to investigate rare disorders and to follow-up a complete birth cohort throughout the entire childhood without selection or attrition biases. In addition, our sensitivity analyses suggest that the findings are robust to some potential sources of bias (see Supplementary appendix). Limitations include, first, that our study is based on real-world clinical diagnoses given in specialized health care, meaning that we have to rely on the categorical diagnoses recorded in the data and have no data on children who are only treated in primary care. This means that service use due to less severe disorders is more likely to be excluded. Therefore, our findings should be interpreted primarily in the context of specialized health care. Second, because child welfare and health care systems vary across countries, generalizations outside Finland should be made with caution. However, our findings seem to reflect international evidence, thus similar patterns are likely to be found elsewhere.

## Conclusion

The majority of children with experience in OHC require mental health treatment, and they comprise a notable proportion of those who are treated for serious and complex psychiatric disorders and self-harm. This necessitates understanding the mental health issues in child welfare services and the social issues in psychiatric services, low-threshold collaboration between child welfare and psychiatric services, appropriately trained staff, and the timely and flexible provision of psychiatric support for children in OHC. Considering that psychiatric and neurodevelopmental disorders are associated with a wide range of immediate and long-term negative outcomes, and that the provision of OHC and psychiatric services is very expensive, developing effective early prevention strategies is essential, specifically for children who exhibit known risks of becoming placed in OHC as adolescents.

## Supplementary Information

Below is the link to the electronic supplementary material.

Supplementary file 1 (PDF 670 KB)

## Data Availability

Access to research data is subject to research permits from the Finnish Institute for Health and Welfare and respective register holders, as mandated by Finnish data protection laws and policies of the register holders.
