# Supplementary material for: The association between treated psychiatric and neurodevelopmental disorders and out-of-home care among Finnish children born in 1997
Source: Eur Child Adolesc Psychiatry. 2021 Jun 8;31(11):1789–98. doi: 10.1007/s00787-021-01819-1 (PMC9666323; doi:10.1007/s00787-021-01819-1)
Supplement: Supplementary file 1 — (PDF 670 KB) [file 787_2021_1819_MOESM1_ESM.pdf]

Supplementary appendix for

‘The association between treated psychiatric and neurodevelopmental disorders and out-of-home care among Finnish children born in 1997’

by

Kääriälä, A., Gyllenberg, D., Sund, R., Pekkarinen E., Keski-Säntti, M., Ristikari, T., Heino, T. & Sourander, A.

SUPPLEMENT TEXT

Search and selection of the reviewed literature

We selected the literature reviewed in this study via the following procedure. We searched several databases for studies published in English, German, Finnish, and Swedish between 1 January 1998 and 3 March 2020 on the prevalence of psychiatric disorders and service use among children in out-of-home care. We searched the following databases: CINAHL; EBSCO: Academic Search Elite; Medline (OVID); Open dissertations; Proquest Sociology Collection (ASSIA, Sociological Abstracts, Social Services Abstracts); PsycInfo; SocIndex; and Web of Science. The terms used in the search are presented in Supplement table 1. We selected relevant literature by scrutinizing the titles and abstracts of the 430 identified studies and by investigating the reference lists of the selected studies.

Additional analyses

We conducted four supplemental and sensitivity analyses. First, we completed the analysis only using inpatient records for the investigated psychiatric and neurodevelopmental disorders. Compared with the main analysis, children in out-of-home care had a higher risk ratio (RR) of being diagnosed with any psychiatric or neurodevelopmental disorder: the *RR* of inpatient care was 6.4 with a 95% confidence interval (CI), 6.1–6.9, versus the *RR* of the main analysis of 3.7 with a 95% CI, 3.6–3.8. This suggests that children in OHC comprise a larger proportion of patients in inpatient care than in outpatient clinics. An analysis of specific disorders is presented in Supplement figures 1 and 2.

Second, we investigated sex differences by running the analysis separately for girls and boys. Both girls and boys in OHC had a similar excess risk of being diagnosed with any psychiatric or neurodevelopmental disorder in comparison to girls and boys never in OHC: the girls’ *RR* was 3.7 with a 95% CI, 3.6–3.9, versus the boys’ *RR* of 3.6 with a 95% CI, 3.4–3.7 (for specific disorders, see Supplement figures 3 and 4).

Third, we checked whether the inclusion of both primary and secondary diagnoses biases our findings and ran the analysis only using primary diagnoses as indicators of psychiatric service use. We found similar results compared to the main analysis: the *RR* of being diagnosed with any psychiatric or neurodevelopmental disorder among children in OHC using primary diagnoses was 3.7 with a 95% CI, 3.6–3.8, versus the *RR* of the main analysis that was 3.7 with a 95% CI, 3.6–3.8 (for specific disorders, see Supplement figures 5 and 6).

Lastly, to address the known issue that some hospital districts have underreported their psychiatric outpatient treatment data [1], we followed the procedure reported by Gyllenberg and colleagues [2] and ran the analysis without these hospital districts. Again, we found similar results compared to the main analysis: the *RR* of any psychiatric or neurodevelopmental disorder diagnosis among children in OHC when problematic hospital districts were excluded was 3.7 with a 95% CI, 3.5–3.8, versus the *RR* of the main analysis that was 3.7 with a 95% CI, 3.6–3.8 (for specific disorders, see Supplement figures 7 and 8).

SUPPLEMENT TABLES

Supplement table 1 The terms used in literature search

Search	Subject terms	Search terms
1.	title	psychiatr* or neurodevelop* or mental* or psychopatholog* or psychological* or emotional* or internalizing or externalizing or aggress* or violen* or "mood disorder*" or "affective disorder*" or substance* or alcohol* or drug* or psychot* or bipolar or depress* or anxi* or panic* or adhd or "attention deficit" or hyperactivity or "learning disorder*" or "learning diffic*" or "learning disabilit*" or "learning problem*" or autis* or asperger* or "behavioral disorder*" or "behavioral disorder*" or "behavioral problem*" or "behavioural problem*" or "behavior problem*" or "behaviour problem*" or "antisocial behavior" or conduct* or defiant or oppositional or "eating disorder*" or anore* or bulimi* or suicid* or self-harm or self-injur* or delinquen*
2.	title and abstract	"foster children" or "foster famil*" or "foster parent*" or "foster care*" or "foster home*" or "foster placement*" or "looked after" or "looked-after" or "out of home" or "out-of-home" or "residential care" or "residential child* home*" or orphanage* or orphan* or "state care" or "public care" or "kinship care" or "children's home*" or placement*
3.	title and abstract	cohort* or prospective or longitudinal or follow-up* or nested or register* or epidemiol* or population* or survey* or "life trajector*" or "life course"
4.	title and abstract	child* or youth or infant* or newborn* or baby or babies or "young people" or "young person*" or adolesc* or youth or teens or teenager* or "early life" or "young adult*"
5.		1 and 2 and 3 and 4

Supplement table 2 STROBE statement

	Item No	Recommendation	Page No
**Title and abstract**	1	(*a*) Indicate the study's design with a commonly used term in the title or the abstract<br>(*b*) Provide in the abstract an informative and balanced summary of what was done and what was found	2
**Introduction**			
Background/rationale	2	Explain the scientific background and rationale for the investigation being reported	3–4
Objectives	3	State specific objectives, including any prespecified hypotheses	4
**Methods**			
Study design	4	Present key elements of study design early in the paper	4
Setting	5	Describe the setting, locations, and relevant dates, including periods of recruitment, exposure, follow-up, and data collection	4–5
Participants	6	(*a*) Give the eligibility criteria, and the sources and methods of selection of participants. Describe methods of follow-up<br>(*b*) For matched studies, give matching criteria and number of exposed and unexposed	a) 4
Variables	7	Clearly define all outcomes, exposures, predictors, potential confounders, and effect modifiers. Give diagnostic criteria, if applicable	5–6, Supplement table 3
Data sources/ measurement	8*	For each variable of interest, give sources of data and details of methods of assessment (measurement). Describe comparability of assessment methods if there is more than one group	4–6, Supplement table 3
Bias	9	Describe any efforts to address potential sources of bias	6, Supplement text
Study size	10	Explain how the study size was arrived at	4
Quantitative variables	11	Explain how quantitative variables were handled in the analyses. If applicable, describe which groupings were chosen and why	6, Supple-ment text
Statistical methods	12	(*a*) Describe all statistical methods, including those used to control for confounding<br>(*b*) Describe any methods used to examine subgroups and interactions<br>(*c*) Explain how missing data were addressed<br>(*d*) If applicable, explain how loss to follow-up was addressed<br>(*e*) Describe any sensitivity analyses	6, Supplement text
**Results**			
Participants	13*	(a) Report numbers of individuals at each stage of study—eg numbers potentially eligible, examined for eligibility, confirmed eligible, included in the study, completing follow-up, and analysed<br>(b) Give reasons for non-participation at each stage<br>(c) Consider use of a flow diagram	6
Descriptive data	14*	(a) Give characteristics of study participants (eg demographic, clinical, social) and information on exposures and potential confounders<br>(b) Indicate number of participants with missing data for each variable of interest<br>(c) Summarise follow-up time (eg, average and total amount)	6–7, Table 1

Outcome data	15*	Report numbers of outcome events or summary measures over time	6–8
Main results	16	(*a*) Give unadjusted estimates and, if applicable, confounder-adjusted estimates and their precision (eg, 95% confidence interval). Make clear which confounders were adjusted for and why they were included<br>(*b*) Report category boundaries when continuous variables were categorized<br>(*c*) If relevant, consider translating estimates of relative risk into absolute risk for a meaningful time period	7–8
Other analyses	17	Report other analyses done—eg analyses of subgroups and interactions, and sensitivity analyses	8, Supplement text and figures
**Discussion**			
Key results	18	Summarise key results with reference to study objectives	8
Limitations	19	Discuss limitations of the study, taking into account sources of potential bias or imprecision. Discuss both direction and magnitude of any potential bias	11–12
Interpretation	20	Give a cautious overall interpretation of results considering objectives, limitations, multiplicity of analyses, results from similar studies, and other relevant evidence	9–11
Generalisability	21	Discuss the generalisability (external validity) of the study results	11
**Other information**			
Funding	22	Give the source of funding and the role of the funders for the present study and, if applicable, for the original study on which the present article is based	1

*Give information separately for exposed and unexposed groups.

Supplement table 3 The ICD-10 codes used for defining the outcome classes

**Diagnostic classes**	**ICD-10**
Any psychiatric or neurodevelopmental disorder	F10–F99
Substance-related disorders	F10–19
Psychotic and bipolar disorders	F20–25, F28–31
Depression and anxiety disorders	F32–F34, F38–F41 (excluding F41.2), F42, F93, F94
Neurodevelopmental disorders	F70–F79, F80–F84, F90, F95
Conduct and oppositional disorders	F90.1, F91, F92
Eating disorders	F50
Self-harm and suicidality	X60–X84, Z72.8, Z91.5, Y87.0

SUPPLEMENT FIGURES

Supplement figure 1 The risks of specific psychiatric or neurodevelopmental disorder diagnoses in inpatient care among children in out-of-home care compared with children never in out-of-home care

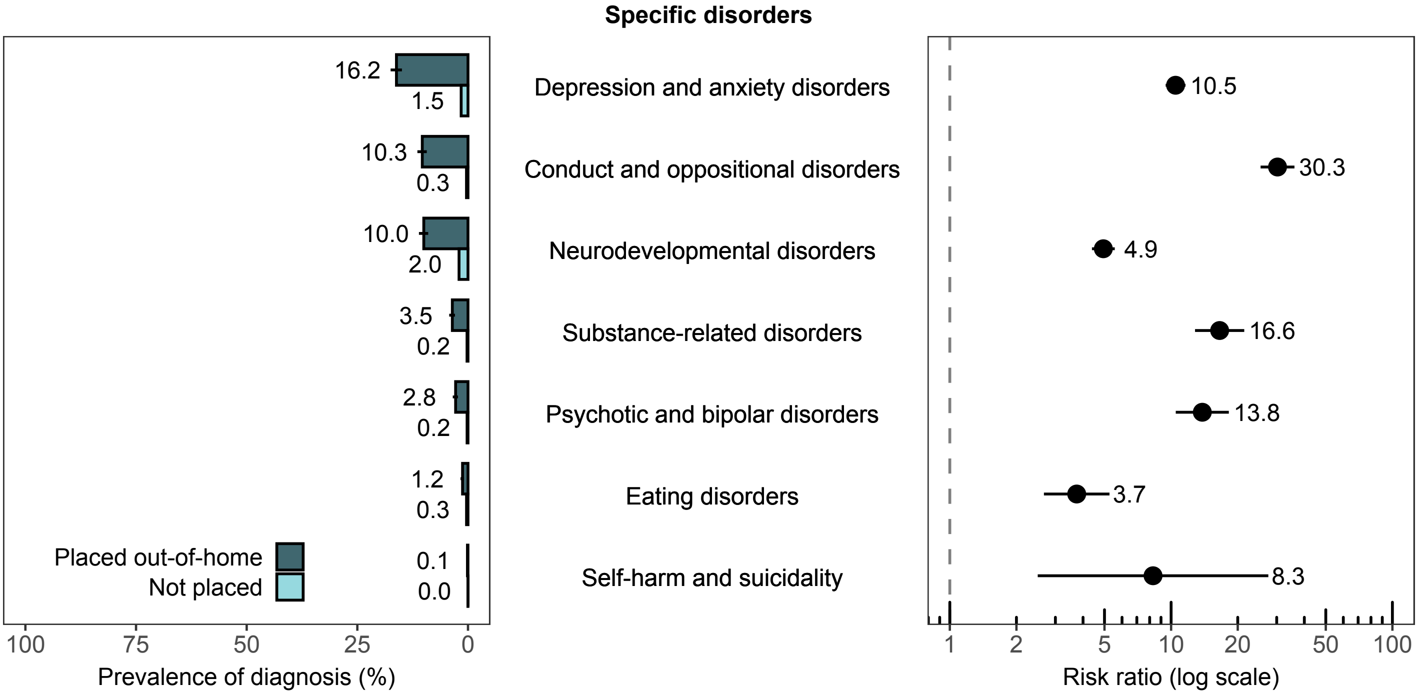

Supplement figure 2 The risk of placement in out-of-home care among children with specific psychiatric or neurodevelopmental disorders diagnosed in inpatient care compared with children without such diagnoses

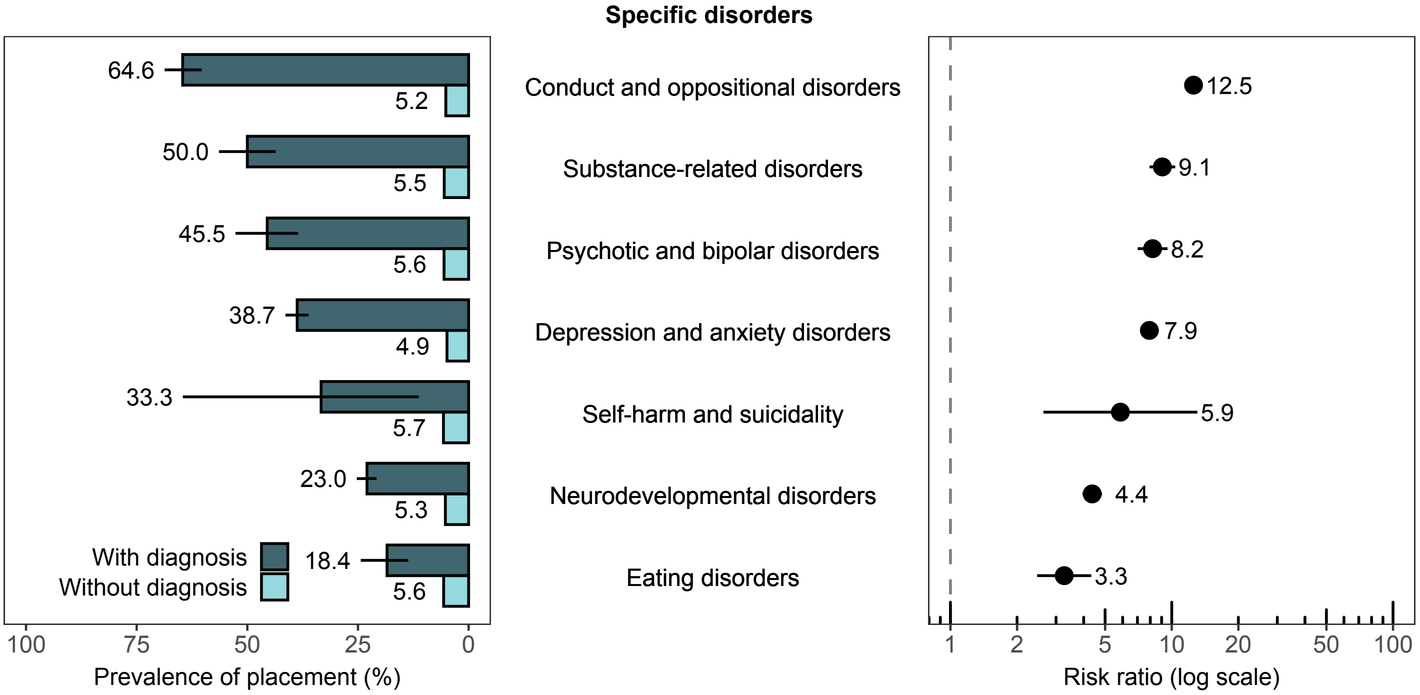

Supplement figure 3 The risks of specific psychiatric or neurodevelopmental disorder diagnoses among girls and boys in out-of-home care compared with girls and boys never in out-of-home care

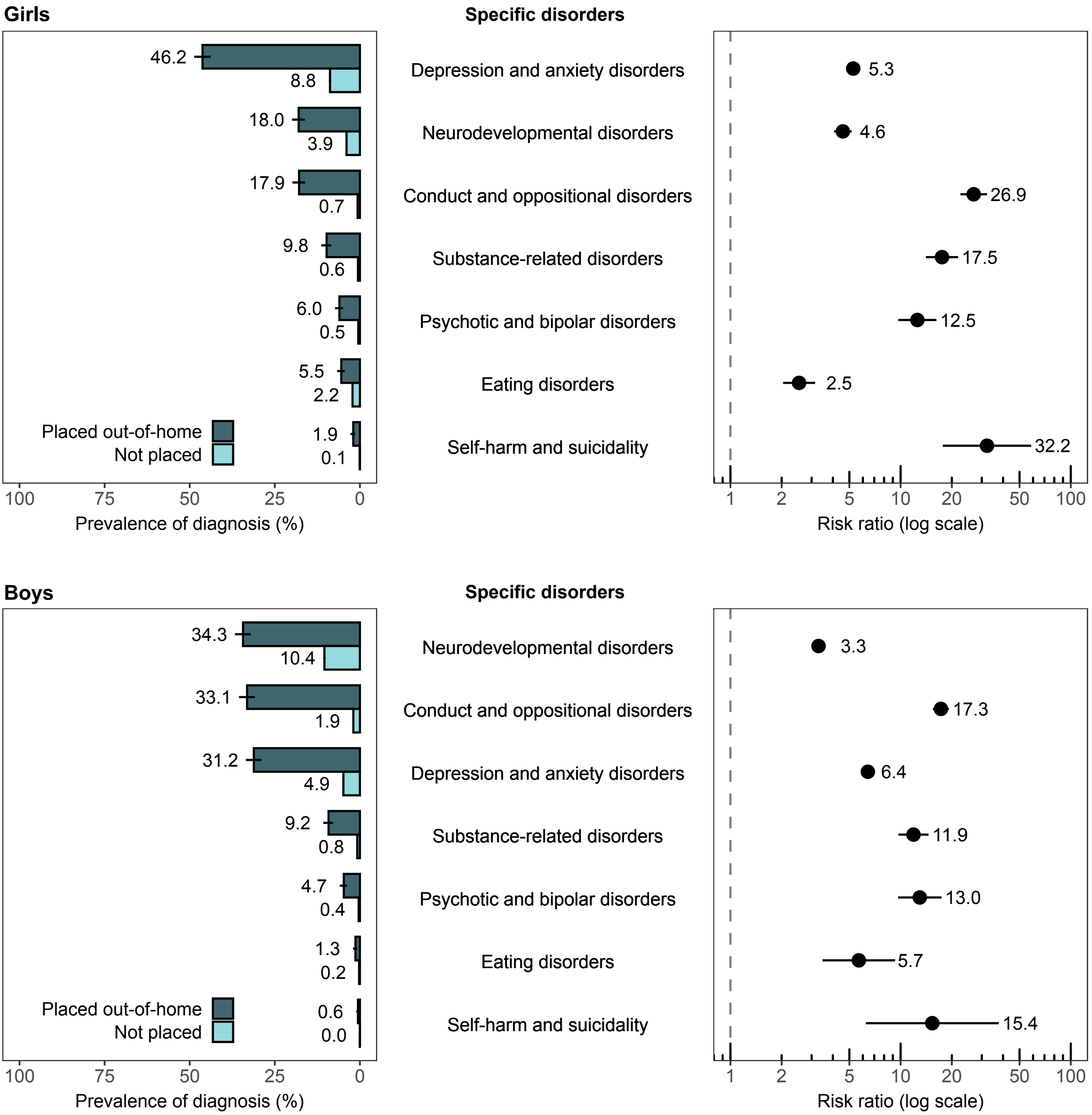

Supplement figure 4 The risk of placement in out-of-home care among girls and boys with specific psychiatric or neurodevelopmental disorder diagnoses compared with girls and boys without such diagnoses

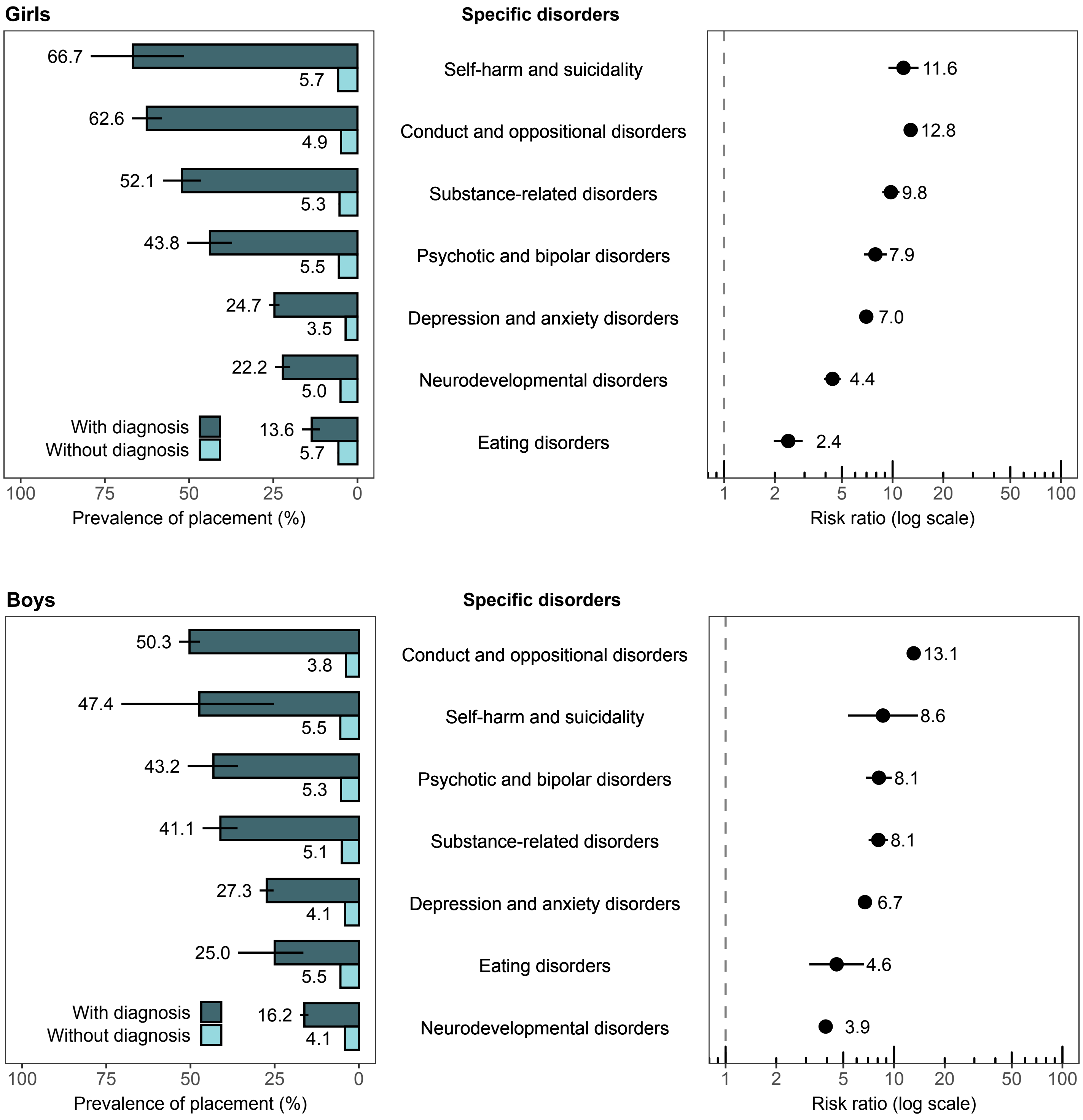

Supplement figure 5 The risks of specific psychiatric or neurodevelopmental disorder diagnoses (secondary diagnoses excluded) among children in out-of-home care compared with children never in out-of-home care

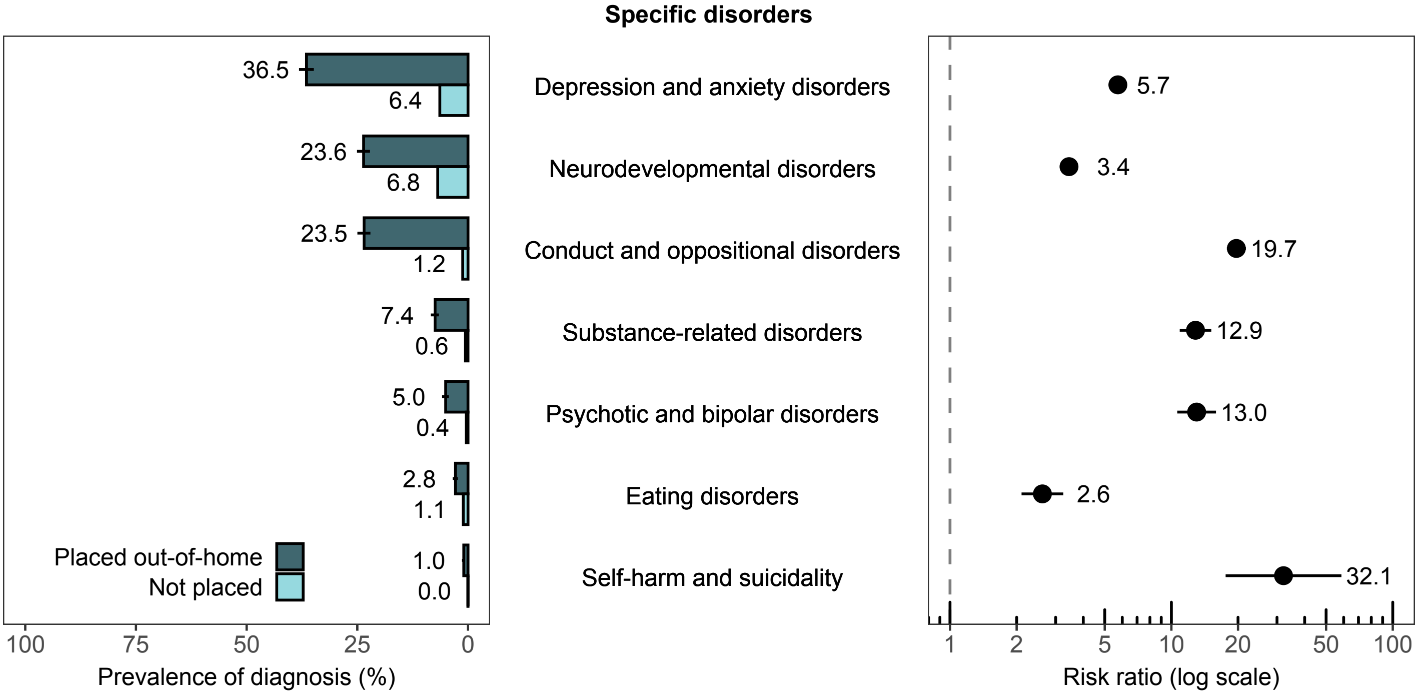

Supplement figure 6 The risk of placement in out-of-home care among children with specific psychiatric or neurodevelopmental disorder diagnoses (secondary diagnoses excluded) compared with children without such diagnoses

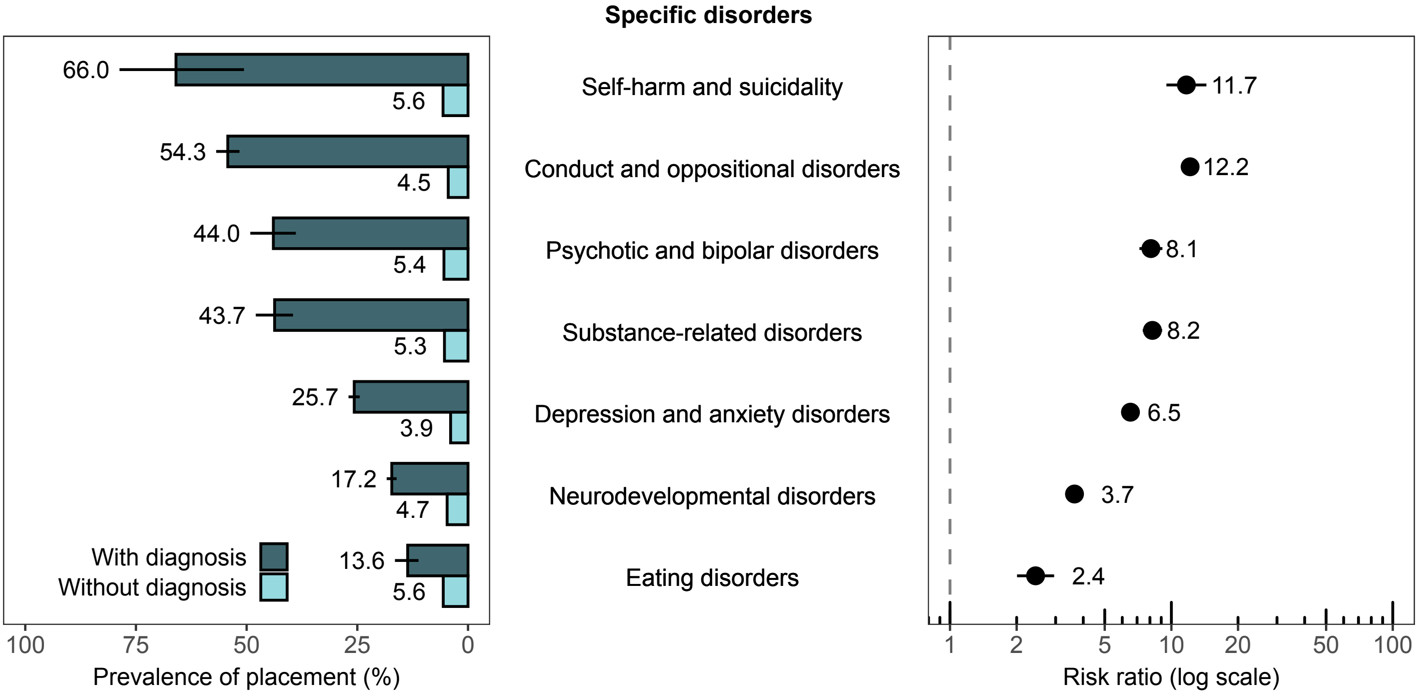

Supplement figure 7 The risks of specific psychiatric or neurodevelopmental disorder diagnoses among children in out-of-home care compared with children never in out-of-home care, excluding hospital districts with reporting issues

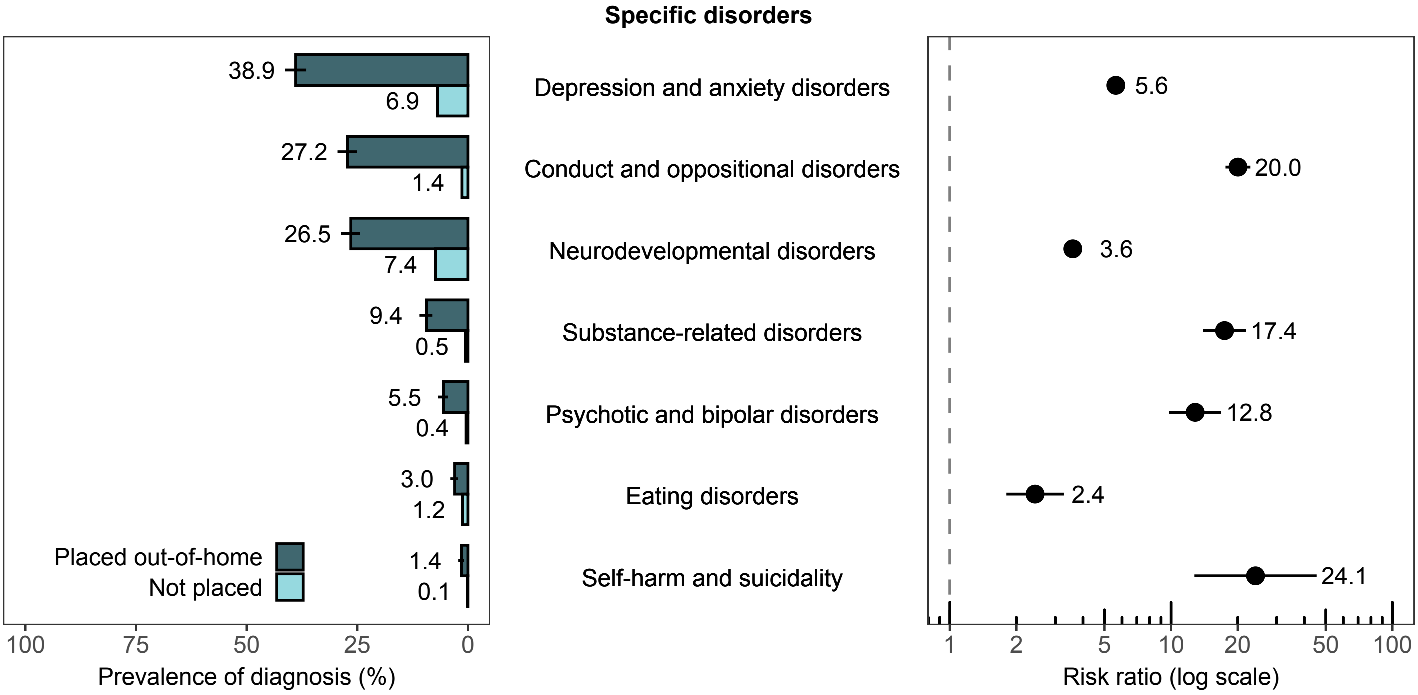

Supplement figure 8 The risk of placement in out-of-home care among children with specific psychiatric or neurodevelopmental disorder diagnoses compared with children without such diagnoses, excluding hospital districts with reporting issues

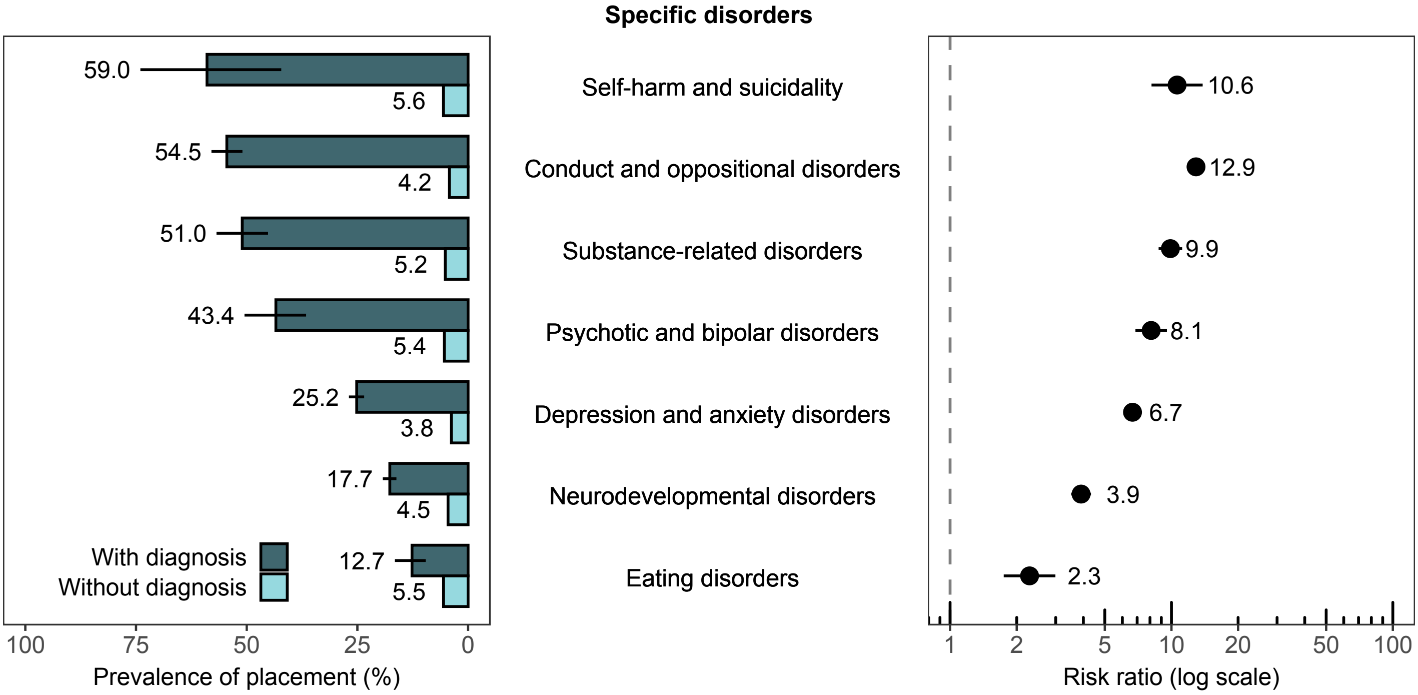

REFERENCES

[1] Sund R (2012) Quality of the Finnish Hospital Discharge Register: A systematic review. Scand J Public Health. 40:505–515. https://doi.org/10.1177/1403494812456637

[2] Gyllenberg D, Marttila M, Sund R, Jokiranta-Olkoniemi E, Sourander A, Gissler M, Ristikari T (2018) Temporal changes in the incidence of treated psychiatric and neurodevelopmental disorders during adolescence: an analysis of two national Finnish birth cohorts. Lancet Psychiatry. 5:227–236. https://doi.org/10.1016/S2215-0366(18)30038-5
